# The DynAIRx Project Protocol: Artificial Intelligence for dynamic prescribing optimisation and care integration in multimorbidity

**DOI:** 10.1177/26335565221145493

**Published:** 2022-12-15

**Authors:** Lauren E Walker, Aseel S Abuzour, Danushka Bollegala, Andrew Clegg, Mark Gabbay, Alan Griffiths, Cecil Kullu, Gary Leeming, Frances S Mair, Simon Maskell, Samuel Relton, Roy A Ruddle, Eduard Shantsila, Matthew Sperrin, Tjeerd Van Staa, Alan Woodall, Iain Buchan

**Affiliations:** 1 Wolfson Centre for Personalized Medicine, 4591 University of Liverpool, Liverpool, UK; 2 Academic Unit for Ageing & Stroke Research, 4468 University of Leeds, Bradford Teaching Hospitals NHS Foundation Trust, Bradford, UK; 3 Department of Computer Science, 4591 University of Liverpool, UK; 4 Institute of Population Health, 4591 University of Liverpool, Liverpool, UK; 5 Public Advisor; 6 4586 Mersey Care NHS Foundation Trust, Liverpool, UK; 7 Civic Data Cooperative, 4591 University of Liverpool, Liverpool, UK; 8 General Practice and Primary Care, School of Health and Wellbeing, University of Glasgow, UK; 9 School of Electrical Engineering, Electronics and Computer Science, 4591 University of Liverpool, UK; 10 Institute of Health Sciences, 4468 University of Leeds, UK; 11 School of Computing and Leeds Institute for Data Analytics, 4468 University of Leeds, UK; 12 Division of Informatics, Imaging & Data Sciences, 5292 University of Manchester, Manchester, UK; 13 Directorate of Mental Health and Learning Disabilities, 8912 Powys Teaching Health Board, Bronllys, UK

**Keywords:** multimorbidity, polypharmacy, frailty, mental health, artificial intelligence, medicines optimisation

## Abstract

**Background:**

Structured Medication Reviews (SMRs) are intended to help deliver the NHS Long Term Plan for medicines optimisation in people living with multiple long-term conditions and polypharmacy. It is challenging to gather the information needed for these reviews due to poor integration of health records across providers and there is little guidance on how to identify those patients most urgently requiring review.

**Objective:**

To extract information from scattered clinical records on how health and medications change over time, apply interpretable artificial intelligence (AI) approaches to predict risks of poor outcomes and overlay this information on care records to inform SMRs. We will pilot this approach in primary care prescribing audit and feedback systems, and co-design future medicines optimisation decision support systems.

**Design:**

DynAIRx will target potentially problematic polypharmacy in three key multimorbidity groups, namely, people with (a) mental and physical health problems, (b) four or more long-term conditions taking ten or more drugs and (c) older age and frailty. Structured clinical data will be drawn from integrated care records (general practice, hospital, and social care) covering an ∼11m population supplemented with Natural Language Processing (NLP) of unstructured clinical text. AI systems will be trained to identify patterns of conditions, medications, tests, and clinical contacts preceding adverse events in order to identify individuals who might benefit most from an SMR.

**Discussion:**

By implementing and evaluating an AI-augmented visualisation of care records in an existing prescribing audit and feedback system we will create a learning system for medicines optimisation, co-designed throughout with end-users and patients.

## Introduction

The Artificial Intelligence (AI) for dynamic prescribing optimisation and care integration in multimorbidity (DynAIRx) project addresses problematic polypharmacy in multimorbidity (co-existence of ≥2 long-term conditions). The aim is to improve holistic care in multimorbidity by supporting medicines optimisation, in alignment with the UK National Health Service (NHS) Long Term Plan and 2021 National Overprescribing Review.^1,2^

As a population we are living longer, driven by medical advances improving survival at all ages.^3^ Age is the dominant risk factor for the acquisition of long-term conditions. The more conditions a patient has, the more associated medications they are likely to take. Polypharmacy describes the use of multiple regular medications by an individual, most often described as taking more than five daily. Without medicines optimisation, polypharmacy may worsen the prevalence, outcomes, experiences and costs of multimorbidity.^4^ The information for coordinating care is hard to assemble and understand, particularly in time-constrained consultations. The effective withdrawal of medications to improve outcomes – deprescribing – is hindered by scattered records impeding the integration of care across providers. Holistic medication reviews have enormous potential to benefit those with multimorbidity, yet there is little support for such reviews. The NHS Long Term Plan^1^ seeks to optimise prescribing, including by deprescribing. Recent evidence has identified limitations in deprescribing during an acute hospital admission, and a proactive, primary care-based approach may be preferable.^5^ Using AI (machine learning for information extraction, dynamic prediction and visualisation), DynAIRx will bring the predictive information and longitudinal care summaries together with guidelines in new visualisations to support medicines optimisation. This combined information will be piloted in prescribing audit and feedback systems that clinicians are using in research and clinical practice.^6^

## Rationale

Despite the need for deprescribing support, evidence of how to do it systematically is lacking. Three Cochrane reviews^7-9^ identified various deprescribing interventions, with barriers to implementation leading to inconsistent effectiveness. Primary-care-embedded development of audit and feedback shows promise for improving prescribing, with success depending on how feedback is delivered.^10^ Previously, such systems have been limited by data supply. The roll-out of integrated/shared care records is now providing the data for patient-centred and locality context sensitive ‘learning systems’.^11^ DynAIRx will develop and implement statistically principled AI approaches to systematically identify problematic polypharmacy in major multimorbidity groups. In order to be effective, AI-augmented feedback to clinicians must be co-produced with clinical stakeholders and reviewed iteratively. Therefore, early engagement with clinicians in the form of a needs analysis will enable:

1. Understanding of the requirements of those involved in SMRs (including patients).
2. Defining the barriers and facilitators to implementation of AI-guided SMRs.
3. Iterative refinement of the proposed prescribing feedback to clinicians.

## Aim(s)

The overall aim of DynAIRx is to develop new, easy to use, AI tools that support general practitioners (GPs) and pharmacists to find patients living with multimorbidity (two or more long-term health conditions) who might be offered a better combination of medicines.

The project will focus on three groups of people at high risk of rapidly worsening health from multimorbidity:

1. People with mental and physical health problems, in whom the prescribing for mental health improvement can lead to adverse physical health consequences.
2. People with complex multimorbidity in the form of four or more long-term health conditions taking ten or more drugs.
3. Older people with frailty as a subgroup of people with multimorbidity at especially high risk of adverse outcomes.

## Objective(s)

The objectives of the DynAIRx project are to:

1. Investigate how SMRs are currently undertaken and what barriers those undertaking them (and the patients in receipt of them) experience.
2. Seek the opinions of key stakeholders involved in the SMR process about the ways in which AI approaches can be used to improve the process and identify what their requirements are for prescriber feedback systems.
3. Identify potential barriers/facilitators to uptake and utilisation of AI-augmented SMRs and audit and feedback dashboards for clinicians.
4. Curate structured clinical data from integrated records (general practice, hospital, and social care) from a variety of NHS Integrated Care Systems covering ∼11m population, adding more structured data from Natural Language Processing (NLP) of psychiatric narratives in Merseyside.
5. Use AI approaches and statistical methods to identify patterns and clusters of conditions, medications, tests, and clinical contacts preceding adverse events across three target groups then build the patterns into biostatistical causal inference and prediction of (clustered) clinical outcomes.
6. Develop visualisation methods for longitudinal summaries of multi-provider care records overlain with risk trajectories, combined with key features from AI-learned patterns/structures and clinical guidelines.
7. Co-design a prototype tool, through iterative review and refinement of feedback systems – participating clinicians, who undertake SMRs will participate in “think-aloud” studies of the protype tool and identify positive and negative features of the tool which will allow the iterative improvement of the prototype (co-developed with patient and public representatives).
8. Refine the later prototypes through user-group feedback and, through two workshops, to explore further the perceived strengths and weaknesses and thus the implementability of the system.

## Methods/Design and Analysis

DynAIRx involves a combination of qualitative stakeholder engagement (DynAIRx Qualitative Phase 1, clinical needs analysis), large-scale health informatics (DynAIRx health data) and co-development/iterative analysis (DynAIRx Qualitative Phase 2) to harness linked data across primary, secondary and social care to create visualisations of patient journeys, risk-prediction estimates and prescribing dashboards to support SMRs. DynAIRx will harness the emerging integrated records mandated for NHS Integrated Care Systems to coordinate services across providers. Through statistically robust approaches, it will predict avoidable multimorbidity and harm resulting from medications.

## DynAIRx Qualitative Phase 1 – Needs analysis and requirements engineering

### Description of study design

**Figure 1.**
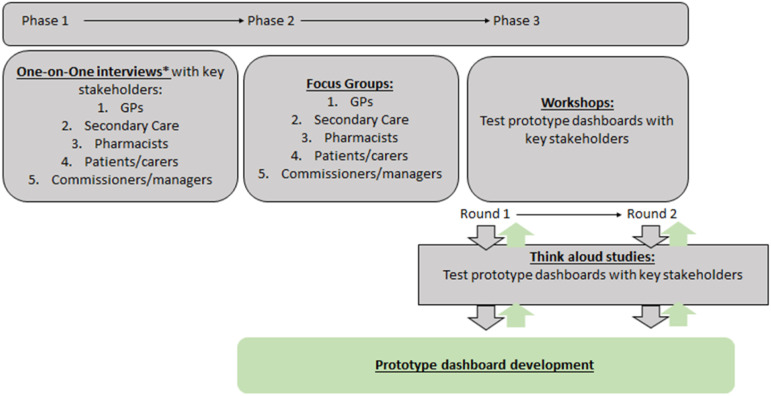
The main flow of work and integration of work-packages (WPs) as a cyclical learning system for medication optimisation in care for people living with multimorbidity and polypharmacy. Each WP will aid in the iterative development of an artificial intelligence (AI) tool via continuous feedback. *One-to-one interviews may continue throughout all phases.

The DynAIRx qualitative studies will explore the perceptions of key stakeholders on how SMRs are currently being undertaken and what the barriers and facilitators are to making them effective and efficient. The research adopts a descriptive and exploratory methodology, and is based on qualitative data from participants regarding their current and retrospective experiences of SMRs (figure 1). This includes semi-structured interviews and focus groups.

The qualitative studies will also explore the opinions of key stakeholders on the prototype prescribing audit & feedback tools that are developed to support SMRs, informed by analysis of patient journeys and AI-assisted integration of care records. This will be undertaken through one-to-one think-aloud studies and mixed-participant workshops.

### Description of study population

Key stakeholder participants involved in the qualitative work include general practitioners, pharmacists, secondary care physicians, patients with multimorbidity and policy makers. Key stakeholder groups are defined in table 1.

**Table 1.** The key stakeholder groups that will be engaged to provide feedback on the current experience of structured medication reviews and to undertake iterative review of prototype prescribing tools.

Stakeholder	Contributors
General Practitioners	General Practitioner trainees, locums, salaried and partner all welcome.
Secondary care clinicians	Specialty input from clinical pharmacologists, psychiatrists and those working in geriatric medicine particularly. Senior trainee and consultant level.
Pharmacists	All grades, spread of primary care and hospital practice/experience
Patient/Carers	Representatives of key target groups including older people (65+) with frailty, multimorbidity, polypharmacy, mental and physical health disorders, caring responsibility for people with complex needs
Commissioner of services/managers/policy makers	This will include representatives from a broad range of professionals working in the design, funding and governance of health services. This includes health & care system wide partners involved in the development of National Health Service England’s Integrated Care Systems – including commissioning, care quality management, health and social care provider, digital infrastructure and population health management perspectives.

### Description of development of research proposal/questions

Research questions of DynAIRx Qualitative–Needs analysis and requirements engineering

1. What are the barriers and facilitators to the uptake and utilisation of an AI-augmented prescribing support system for SMRs from the perspective of primary and secondary care clinicians, pharmacists, patients, and commissioners/managers involved in SMR services?
2. What are the features that would make such a resource acceptable and usable?

## Interventions and comparisons

### DynAIRx Qualitative In-depth Interviews

Semi-structured one-to-one interviews will be undertaken with a broad range of representatives from Primary Care Networks, GPs, pharmacists (primary care and chief), clinical pharmacologists, practice managers and patients to understand their priorities for such reviews and potential barriers/facilitators to implementation.

Semi-structured interviews will allow us to elicit participant personal feelings, opinions, and experiences, and help the researchers to gain insights into barriers and facilitators to future uptake of the proposed systems.

## DynAIRx Qualitative **Focus groups**

Semi-structured interviews will be followed by broader focus group discussion (1-2 groups) across the 5 stakeholder groups (4-8 participants per focus group). The total number of participants will depend on the themes that emerge and the requirement for further exploration. Focus group interview guides will be co-developed to address the key research questions, enhanced by themes that emerge from the initial one-to-one interviews.

Prescribers’ requirements for support with SMRs will be identified across the key groups. This will include insight into data-driven medication reviews, including what clinicians consider high-risk vs high-volume prescribing. Work will also focus on clinical uncertainty. For example, exploring prescriber needs in high-risk situations, such as severe mental illness, where stopping an antipsychotic may not be viable, yet the dose could be adjusted to a safer level, or an alternative drug with lower cardiovascular risk could be prescribed. Discussions will explore how medications might be best prioritised for older people living with frailty, and people with complex multimorbidity, including using the The National Institute for Health and Care Excellence (NICE) Database of Treatment Effects and Scottish Polypharmacy Guidance.

Task-based workshops/focus groups may be supported by ongoing, semi-structured qualitative interviews with stakeholders, including Clinical Commissioning Group Leads and Chief Pharmacists, alongside patients and carers across our key groups. All workshops/interviews will be audio-recorded, with participant consent, and transcribed for thematic analysis.

## Description of sample selection/data collection

Semi-structured interviews (∼10-20 participants across 5 stakeholder groups) and focus groups (1-2 per stakeholder group involving 4-8 participants) will be undertaken via video conference (or telephone for one-to-one interviews). The groups are deliberately small to engage effectively and allow for open discussion and to obtain the views of a broad range of practitioners nationally to examine the current scope of practice. Participants will contribute their expertise from community, primary and secondary care practice.

## Recruitment

### Inclusion criteria

*Patient and carer representatives:*

1. Individuals with (or carer for someone with) any of the following criteria:
  a. Multiple (4 or more) long term health conditions.
  b. Co-existing mental and physical health problems.
  c. Prescribed ≥10 regular medications.
  d. Frailty.
2. Age 18 or over.

*Health care/management professionals:*

Health care or management professionals (including doctor, pharmacist, nurse, commissioner of clinical services, manager of clinical services):

1. Working in health care setting where review of prescription medications is a regular part of the clinical workload.
2. Working in key stakeholder groups including any of:
  a. General practice.
  b. Secondary care (geriatric medicine, clinical pharmacology, falls clinics, mental health practitioners).
  c. Clinical commissioning of services or management of clinical services (practice managers).
  d. Pharmacist (primary care ideally).

### Exclusion criteria

*Patient and carer representatives:*

• Unable to give informed consent to participate.
• History of hearing or speech impairment to a degree that would render normal conversation impossible via video interview and this is their only option; however, all participants who have such impairments who wish to participate would be offered the option of a face-to-face meeting, along with necessary adjustments, to ensure inclusivity.
• Unable to communicate in English.

*Health care professionals:*

• Not involved with prescribing

Patient and carer participants will be recruited via a variety of networks including:

*•* Outpatient clinics for long-term conditions.
• Via social media (Facebook, Twitter) email to the qualitative team.
• Via networking at events (conferences, public engagement etc).
• Sampling the CARE75+ cohort participants with frailty, at the University of Leeds. Frail participants are defined using either the phenotype model, or as having mild/moderate/severe frailty using the electronic Frailty Index.^12^ The research team will be provided with restricted details (e.g. name, telephone number, address) of CARE75+ participants who meet the eligibility criteria by the CARE75+ study team. Only CARE75+ participants who have already given consent to be approached for future research studies, including provision of this restricted data, will be approached. Study information will be mailed to the potential participant by the research team, who will subsequently contact the potential participant to discuss the study and whether they are interested in being involved.
*•* Charities including Age UK and Mind. Working in partnership with the charity, patient information sheets will be sent to charities for distribution through their networks.
• Mental health directorate expert patient reference groups and patient liaison group to engage service users.

## Qualitative Data collection

### Interview format

All interviews will be audio-recorded, with participant consent, and transcribed for thematic analysis.

• Individual one-on-one semi-structured interviews will be conducted over telephone or video conferencing with 1-2 members per key stakeholder group (GPs, secondary care, commissioning/management of services, pharmacists, patients/carers).
• Demographic (name of surgery, Trust or Clinical Commissioning Group, grade of profession) and professional information will be collected from Health Care Professionals (HCPs) (e.g. hospital registrar or consultant, GP registrar, locum, partner, pharmacist years of experience, years undertaking medication reviews) prior to starting the interview.
• Prompt questions as per interview guide (Supplementary Appendix 1).

An interview guide (Supplementary Appendix 1) will be co-created with experts by experience (professionals and PPI) focused around key areas of interest including:

• What data do prescribers/practices need to undertake effective Structured Medication Reviews efficiently?
• How are Structured Medication Reviews currently being undertaken, by whom,where, and how long do they take?
• What kind of digital tools and supports will be most useful?
• What do participants consider the top priority target medication challenges relating to key multimorbidity groups (older people with frailty; co-existing physical and mental health problems; complex multimorbidity and potentially problematic polypharmacy)?
• What are likely barriers/facilitators to uptake and utilisation and sustained use of AI(-augmented) tools?

### Focus group format

Initial semi-structured interviews will be followed by broader focus group discussion across the stakeholder groups depending on the numbers attending each focus group, the themes that emerge and the requirement for further exploration. Focus group interview guides will be further developed from the key questions, enhanced by themes that emerge from the initial one-to-one interviews.

• Each key stakeholder group (GPs, secondary care, commissioning/management of services, pharmacists, patients/carers) to contain approximately 4- 8 participants.
• Demographic information will be collected prior to the focus group from participants.
• Minimal information will be collected from HCPs (name of surgery, Trust or Clinical Commissioning Group (CCG), years trained, grade (e.g. hospital registrar or consultant, GP registrar, locum, partner, pharmacist years of experience, years undertaking medication reviews) prior to starting the focus group.
• The co-produced topic guide will be followed to structure the focus group (Supplementary Appendix 2).
• Digitally recorded.

## DynAIRx health data

### Description of study design

Machine learning algorithms will be used to bring the predictive information and longitudinal care summaries available in integrated care records together with guidelines in new visualisations to support medicines optimisation. This combined information will be piloted in prescribing audit and feedback systems that GPs are using in research and practice.^6^ DynAIRx will develop tools to combine information from electronic health and social care records. De-identified patient data obtained from health records will be combined with clinical guidelines and risk-prediction models to ensure that clinicians and patients have the best information to prioritise and support Structured Medication Reviews.

AIs will be developed that combine information from multiple records and guidelines and calculate risks of hospital admissions and other adverse outcomes for our three multimorbidity groups. To ensure this information is easily understandable, visual summaries of patients’ journeys will be developed, showing how health conditions, treatments and risks of future adverse outcomes are changing over time. These visual summaries will be tested in general practices across northern England and improved based on feedback from clinicians and patients (described in DynAIRx Qualitative Phase 2).

## Description of development of research proposal/questions

### Research questions of DynAIRx Health Data

1. What combinations of diseases, medications, investigations and clinical contacts are associated with the greatest degree of adverse outcomes in patients with high risk of harm (frailty, co-existent mental and physical health problems and complex multimorbidity)?
2. Can patient journeys over time and across care providers be adequately visualised in the context of clinical guidelines and be enriched with causal inference methods?
3. Can a learning system be created that incorporates the needs of prescribers and patients alongside the key high-risk trajectory indicators?

## STRUCTURED CLINICAL DATA AND NARRATIVE PROCESSING

### Description of study population

Datasets will be created within Trusted Research Environments (TREs), making clean data available for further analysis. A federated approach to clinical data will enable access to structured data from across Cheshire & Merseyside (Combined Intelligence for Population Health Action, CIPHA, platform), Greater Manchester, and Yorkshire & Humber, covering a population of ∼11m from existing regional shared care record systems, which provides research access and prescriber audit and feedback.

### Description of sample selection/data collection and curation

Core research datasets will be curated and maintained from these integrated general practice, hospital, and social care records, where available. Accredited (ISO27001/NHS DSPT) cloud-based TREs support the software, tools, compute, and governance for research access. These federated data sources will feed a minimum core dataset (MCD) for evaluation and deployment – including coded data fromgeneral practices as well as Secondary Uses Service data from hospitals and structured community and mental health datasets, where available.

The MCDs will be extended, where available, with information extracted from, and tracked across, clinical narratives using NLP-contextualised language models such as BERT. This builds upon existing healthcare NLP applications and annotated datasets, such as WEB-RADR (extracting events related to adverse drug reactions)^13^ and AVERT (mining mental health narratives from clinical letters).^14^ The data include over four billion annotations over 12 years in a large mental health and community provider trust, plus inputs from other regions. To extract (de)prescribing events, related drugs and contexts across narratives will be identified. This involves named entity recognition for detecting drug name or label variations; context extraction, such as treating an adverse effect of another drug; and entity mapping across time and/or sources, including extraction of time references for tracking prescribing journeys. These data can then be linked and validated against the routinely collected and integrated care record data.

A data catalogue will be maintained. A federated and open-source approach will be taken to data analyses – sharing all code via a GitHub public repository.

## STATISTICAL LEARNING AND CLUSTERING FOR MULTIMORBIDITY PREDICTION

### Interventions and comparisons

The structured data that have been curated and processed will be analysed to discover clusters of multimorbidity and polypharmacy with high apparent prescribing harm in the key multimorbidity groups. Machine learning and statistical methods will be used to develop prediction models for adverse outcomes, and to estimate which patients may benefit most from a structured medication review.

Adverse outcomes may include events such as falls in older people with frailty; strokes in people with severe mental illness, diabetes, and hypertension; and hospitalisation for adverse drug reactions or emergency/unplanned hospitalisations. Patterns indicating adverse outcomes or sentinel events such as prescribing cascades will be extracted from the curated data. Patient histories will be modelled as temporal graphs capturing clinical events (diagnoses, prescriptions etc.) in their timeline, and extracted using 3D convolutional neural networks. This will exploit recent advances in video and time-series classification to discover temporal patterns and not just sequences of events (as with recurrent neural networks). The output will be a time-series of clinical feature vectors, which can be used to predict outcomes or to define clusters of typical patient trajectories. Soft, temporal clustering algorithms will be used to track a patient’s membership of each cluster over their recorded history. For instance, they may move gradually from a low-risk cluster to a cluster with high risk of hospitalisation. The identified patterns/clusters will be visualised and user feedback (described in DynAIRx qualitative Phase 2) used to refine the AI (e.g., find clusters that deviate from NICE guidelines).

The distilled patterns/clusters will be used to generate hypotheses, followed by development of explainable information. To reduce the risk of posing spurious associations (e.g., confounded relationships) as causal relationships, an expert panel will be called upon for potential reference. Where available, causal estimates will be derived from randomised controlled trials, or other robust external sources such as Mendelian randomisation studies. When required, causal estimates may be derived from the data in-hand using g-methods.

Visualisation and expert clinical and evidence-based reasoning are key in 1) informing the construction of graphical models to represent causal relationships between variables, and 2) weighing the plausibility of identified putative causal relationships. Where a causal relationship is in doubt, it will be examined within the key stakeholder groups (described in table 1), requesting additional data curation as needed.

In parallel, dynamic clinical prediction models will be developed to identify risks of adverse outcomes and expected multimorbidity trajectories. These can be aggregated to practice level to enable identification of clinicians/practice outliers to better guide supportive interventions. The incorporation of causality then enables the identification of clusters/individuals at high risk, and prioritises those where the identified causal pathways suggest that structured medication review might benefit the patient(s). The models also form a strong basis for future work to identify anticipated benefits (effect sizes) of potential interventions such as deprescribing at an individual patient level. In principle, such tools can be used to support clinicians performing medication reviews (as well as suggesting which patients/clusters can benefit from medication reviews, as proposed here), as risks of multiple outcomes can be evaluated and discussed under different intervention strategies.

Particular attention will be paid to explainability of AI, focusing on feature importance, rule extraction and consistency in individual risk prediction between AI models with comparable population-level performance. In contrast to ‘black box’ AI approaches to prediction, the methods utilised here are anchored in causal inference, explicitly handling causality. Causal queries are used to generate predictions under hypothetical interventions, which naturally ensures model explainability. Explainability and temporality are also embedded in the clustering approaches (describing the temporal characteristics of individuals within each cluster), and the visual summaries (visualizing patients over time and between clusters). Data sparsity is explicitly represented as uncertainty within directed acyclic graphs prompting requests for further data or experimentation. Counterfactual causal reasoning will be used to identify and minimise possible biases and unfairness in our models.

### Mitigation of bias

Bias due to confounding factors (especially socio-economic and demographic) and data quality will be mitigated via a systematic bias assessment as part of the statistical learning and clustering for multimorbidity prediction. The consistency of AI results in individual predictions for models with comparable population performance will be evaluated and the effects of hyperparameters explored; models with acceptable hyperparameters can yield varying individual predictions.^15^ This methodology will consider how risk predictions vary between clinical sites (as reported for QRISK, a widely used risk prediction tool^16^).

## COMBINED LONGITUDINAL DATA VISUALISATION FOR MEDICATION REVIEWS

Creating visual summaries has four stages. First, implementing functionality to extract and aggregate prescribing/disease events at cohort/patient and longitudinal/cross-sectional/overall granularities using curated data. This will provide a stable application programming interface (API) to connect care record systems to DynAIRx prescriber dashboards, in order to detail the ‘chronicles of events’ identified by the key stakeholder groups in DynAIRx qualitative.

Second, exploring alternative approaches for presenting interactive visual summaries of prescribing and disease events. Standard single-screen dashboards will provide a baseline but are unlikely to satisfy GP/pharmacist’s requirements. Exploring dashboard designs from two approaches better suited to multifactor, temporally complex data: 1) dashboard networks, where dozens of types of events of interest are summarised in a miniature dashboard, which are connected in a network to portray temporal changes between patients/cohorts^17^; and 2) the QualDash engine, already deployed in cardiology and paediatric intensive care (five hospitals).^18^

Stage 3 will compare the pros and cons of the alternative approaches with GP/pharmacist end-users, selecting and then implementing the best approach (detailed in DynAIRX Qualitative Phase 2). This stage will incorporate data generated by statistical learning and clustering to provide visual summaries and drill-down of patient histories in the context of patient clusters, trajectories, drug-drug interactions and clinical guidelines. It will also provide customisable functionality needed to present the patient event summaries in the context of feature spaces from the statistical learning output, which will be invaluable for: (a) identifying features that distinguish one step from the next in patients’ journeys, and clusters of patients from each other, and (b) gaining clinical input about the explainability of the models.

The final stage, evaluation, includes the development of a user guide and quick start tutorial, and hands-on evaluation with GPs/pharmacists performing Structured Medication Review scenarios (covered by DynAIRx qualitative protocol Phase 2).

## PRESCRIBER FEEDBACK AND LEARNING SYSTEM – Data analysis

Translation of research findings into daily clinical practice is a major challenge. There is considerable need for clinical decisions to be based on the best available evidence, but often this evidence is not available (no trials conducted) and guidelines are only generic and usually relate to single conditions. It also needs to be balanced against clinician and patient/carer choice and preference, affordability according to local formularies, and congruence about goals and management plans between professionals and patients/carers to enhance shared agreement about treatment regimes.

The Learning Healthcare System has been proposed to better integrate research and clinical practice.^19^ This approach involves iterative phases including data analytics (data to knowledge), feedback to clinicians (knowledge to performance) and implementation of quality improvement activities by the clinicians (performance to data). The cycle of the Learning Healthcare System starts again by evaluating the effectiveness of these quality improvement activities. The analytics phase includes a detailed data analysis of the opportunities and challenges in current clinical practice and the local site (including analysis of the effectiveness of current activities). The results of the analysis would enable identification of care pathways and conditions ripe for focused targeting for improvement. The second phase involves review by the clinicians of these results and decide which have sufficient credibility to generate recommendations for change, ideally customized to its own specific circumstances. The third phase involves implementation of these recommendations by clinicians. Cluster trials have reported that data feedback can be effective in optimising prescribing.^20^ The effectiveness of data feedback has been found to depend on content and how the feedback is provided including visualisations.^10^ Feedback on simplistic targets may lack effectiveness (an example is the Quality and Outcome Framework that only resulted in small improvement despite its major investment.^21^ Engagement with clinical stakeholders in the developing of feedback prototypes and iterative reviews are important in improving the feedback effectiveness. The Learning Healthcare System approach can also tailor feedback to individual clinical sites, prioritising to the most frequent challenges, as well as tailor feedback to care practices with best outcomes as determined by e.g. statistical learning. Furthermore, technologies that are most successful in optimising professional practice are those that explicitly use behaviour change techniques in their implementation including peer-to-peer comparisons.^22^

Analyses in large research datasets (including > 5 million patients aged 65+) are ongoing. AI approaches found that medication patterns were strongly associated with ADR-related hospital admission (Odds Ratios [OR] of 7) and emergency admission (ORs of 3). Analyses of multiple drug-drug interactions with antibiotics (as listed in the British National Formulary) are providing information on relative as well as excess absolute risks. Analyses of medication reviews in polypharmacy patients found limited changes in prescribing in before-after analyses, highlighting the need for better evidence and support. Techniques such as random forest and gradient boosting methods will be used in this project to identify challenges and higher rates of adverse outcomes in medicine combinations used by our study populations. This will be followed by practice and peer comparisons^23^ to identify possible areas of improvement, which could be used in the feedback to practices.

## PRESCRIBER FEEDBACK AND LEARNING SYSTEM – Dashboard co-development

A recent BRIT2 clinical pharmacist (CP) workshop examined analytics-based input to support Structured Medication Reviews (SMR) for polypharmacy patients. CPs were interested in analytics which indicate the clinical risk of BNF drug-drug interactions, identify problematic prescribing patterns in the community (e.g. unexpected psychopharmacological effects), and target medication reviews toward high-risk patients. CPs felt that they were currently overloaded with information and popups as existing systems did not fit with the way they work. They were very clear that any tool would need to be very well targeted, user-friendly and have good explainability, which is very important as CPs must rationalise medication changes with other clinicians and patients and cannot ‘just trust the data’.

Prescribing dashboards to support SMRs will be co-developed with key stakeholder groups and deployed in an existing prescribing audit and feedback used by GPs.^24,25^ Participating clinicians, who undertake Structured Medication Reviews in Liverpool, Manchester, Leeds and Bradford, will receive novel reports to support reflective practice concerning their patients with notable multimorbidity and polypharmacy issues in our key areas of study. The reports will extend the BRIT2 platform.^11^ BRIT2 includes general practices in northern England. Technical specifications have been agreed for embedding/enhancing BRIT2 in the Graphnet Integrated Care Record System as part of the CIPHA expansion programme, which currently covers North West England and parts of the Midlands and South England. Data will be analysed in the TREs and the results fed back to practices via practice-specific dashboards.

Patterns of conditions, medications, tests, and clinical contacts antecedent to the multimorbidity events uncovered and the novel visualisations created will be incorporated into prescriber dashboards. DynAIRx qualitative engagement will help shape this content into forms that clinicians and practices find useful. Variability in multimorbidity-related prescribing across practices/prescribers will be studied as part of this. This will build on BRIT2 which is currently analysing large cohorts of elderly patients with national primary care data extracts (Clinical Practice Research Datalink, Aurum). These results will be used for benchmarking under existing ethics approvals. Each practice population of multimorbid patients will be matched by propensity for adverse outcomes, morbidity cluster and data quality. This matching helps show where a practice deviates from its peers. As part of DynAIRx qualitative engagement, clinicians will be able to comment on dashboards, providing feedback to researchers on the acceptance of the results. The applicability of social-norm, practice/prescriber-level feedback to medicines optimisation in multimorbidity will be studied with key stakeholders, with particular consideration of the scale achievable at low cost through AI.

Analyses for each iteration of feedback will be prioritised by users (DynAIRx qualitative). A particular focus will be quantification of the absolute risks of interactions and, where possible, presence of effect modifiers (such as level of polypharmacy).

At least two cycles of updating practice-tailored dashboards will be applied (DynAIRx qualitative). The effects of the feedback will be studied within statistical learning and clustering for multimorbidity prediction using interrupted time series models and recurrent neural nets.

*Research questions of DynAIRx Qualitative Phase 2 – Prototype iterative analysis*

1. What are the strengths and weaknesses of the AI-augmented prototype dashboard and prescriber reports?
2. What improvements could be made to ensure the AI-augmented process achieves maximal clinical utility?

## DynAIRx Qualitative Phase 2 – co-development/iterative analysis

### Think-aloud study format

Two rounds of one-to-one ‘think-aloud’ studies on prototype systems will be undertaken with a small group of clinicians to understand perceived strengths and weaknesses of the prototypes and to iteratively refine them. Participants will be asked to comment on components of the systems, with prompts and questions to elaborate responses. Participants will be encouraged to suggest improvements and explain what they like/dislike, which aspects are (not) intuitive, and how they envisage using such systems in real-life. Findings will be shared immediately with dashboard developers to refine prototypes ahead of the next think-aloud study.

Approximately 10 think-aloud studies are planned across a variety of potential users. They will be recorded and transcribed and the transcripts thematically analysed. Data relating to implementation will be conceptualised through a Normalization Process Theory (NPT) lens. Comments will be noted to be either positive, where the user liked or identified with what they saw, or negative where the user disliked or disagreed with what they saw, or where the user suggested improved content, presentation, or interaction.

• Each think-aloud study will consist of one participant, and will take approximately 2 hours.
• Approximately 4-6 studies will occur per iteration of the resource.
• Participants will be given a brief task sheet for them to work through utilising aspects of the online resource/dashboard, taking approximately 2 hours.
• Participants will be asked to talk through what they are doing as they are completing the task sheet.
• Following this, participants will be asked to provide any general thoughts or feedback from their interaction.
• Think-aloud studies will be audiotaped and transcribed to ensure no feedback is missed.

### Task-based workshops format:

Stakeholders will also critique each major new version of the system in two workshop events – one for each development iteration. Emerging findings will be shared with the health data analysts, ensuring that statistical learning and visualisation are informed by clinician, commissioner and patient insights. Following the development of the final DynAIRx prototypes, we shall present them to the wider group for feedback to enable further discussion of perceived strengths and weaknesses and to address future implementability.

We will audio record and transcribe the sessions and thematically analyse transcriptions as described earlier. Comments will be noted to be either positive, where the user liked or identified with what they saw, or negative where the user disliked or disagreed with what they saw, or where the user suggested improved content, presentation, or interaction.

Think-aloud studies and workshops will be organised face to face, ideally in the practitioner's own place of work where possible and practical to obtain the most real-world usage data. However, these could also be undertaken remotely if felt appropriate, for example, if pandemic restrictions were to be re-introduced or at the preference of participants. Both primary and secondary care practice will be covered.

### Organising the qualitative data

The recording of the interviews will be transcribed and anonymised (all names and other identifiable information will be removed). The digital recordings will be held securely at the University of Liverpool or University of Glasgow, with secure file transfers to/from the transcription company. Once the transcripts are checked against the audio files, those audio files will be deleted.

The socio-demographic information, including information on HCP roles, of the interview and focus group/workshop and think aloud study participants will be entered into a spreadsheet and then exported into NVivo software to create case nodes. Tables will be constructed summarising the socio-demographic and role data. The case nodes will facilitate the comparability of themes within and between groups and across the different study contexts.

### Thematic analysis of data and normalisation process theory (NPT)

All semi-structured interviews, focus groups, think-aloud studies and task groups will be audio-recorded and transcribed verbatim to form the data for analysis. Transcripts will be read and re-read and a thematic analysis will be undertaken using Braun and Clarke's six step framework for thematic analysis which combines elements of deduction and induction whereby some themes are expected to be found in the data based on the literature or the theoretical framework (in the case of think alouds and task-based workshops reviewing prototypes that will be Normalization Process Theory) and others appear by themselves during analysis.^26,27^

The six steps are: familiarization, coding, generating themes, reviewing themes, defining and naming themes, and writing up^26,28^ This approach essentially involves an exploration of the data to identify patterns, themes and/or theoretical constructs. This involves detailed reading of the transcripts and identifying all key issues, concepts and themes, drawing on a priori issues while being alert to new ideas raised by the participants.

This work will help us understand stakeholder priorities for SMRs and potential barriers/facilitators to implementation. Once themes are finalised, they will be mapped onto the constructs of NPT: coherence (sense making); cognitive participation (engagement work); collective action (operationalisation work); and reflexive monitoring (appraisal), where appropriate. The data will not be forced to fit the constructs of NPT. NPT will instead be used as a theoretical lens with which to interrogate the findings.^27,29^ NPT has been widely used to consider how individuals and groups understand, integrate, and sustain digital or new ways of working (e.g. SMRs) into everyday practice, and has enhanced (understanding of) implementation processes.^30^

Data analysis will be carried out by the DynAIRx clinical researchers and the post-doctoral research assistants (PDRAs). Coding clinics will be undertaken to refine the themes identified and ensure consistency of coding across the team. A common analytical framework will be developed to ensure consistency in analysis across the various study locations. The analytical framework would be flexible and iterative and continuously refined as the analysis evolves. NVivo software will be employed to organise the data, and help manage the data analysis process. All the DynAIRx clinical researchers will be trained in how to use the software. Data analysis will be undertaken in parallel with data collection. This will help the researcher determine whether saturation has been reached on any of the research questions and to identify gaps for further data collection.

Any quotations used in any reports will be anonymised.

## Ethics approval and dissemination:

The study has been approved by the Newcastle North Tyneside Research Ethics Committee (REC reference:22/NE/0088). No safety concerns were identified. Study findings will be presented at public meetings, national and international conferences and published in peer-reviewed journals.

## Discussion and Conclusion

DynAIRx will provide patient benefit by: a) targeting medication reviews/optimisation to those most at risk from harm due to problematic polypharmacy and most likely to benefit from SMR; b) reducing the risks of drug-related harms; c) freeing up clinician time for patient interaction through automated data collection for structured medication reviews; and d) providing a clear, visual summary of disease trajectories to inform clinician/patient discussion.

Key outputs from DynAIRx (mapped to objectives and research questions, table 2) include:

1. Evaluation of key challenges and opportunities around medicine optimisation in general practices
2. A pipeline of structured and unstructured care data into multimorbidity (AI) research.
3. An AI framework for identifying those most at risk of problematic polypharmacy and for discovering disease trajectories that should trigger high priority SMRs.
4. Novel visualisations of patient journeys enhancing medication reviews.
5. Integration of outputs 1 – 4 to produce a clinically useful learning healthcare system, co-developed by the end users and supporting the delivery of SMRs by GPs and pharmacists and be accessible to patients/carers.

**Table 2.** Table detailing how the study objectives combined with research questions lead to the key outputs.

	Objectives	Research questions	Key outputs
1	Investigate how SMRs are currently undertaken and what barriers those undertaking them (and the patients in receipt of them) experience.	What are the barriers and facilitators to the uptake and utilisation of an AI-augmented prescribing support system for SMRs from the perspective of primary and secondary care clinicians, pharmacists, patients, and commissioners/managers involved in SMR services? What are the features that would make such a resource acceptable and usable?	Evaluation of key challenges and opportunities around medicine optimisation in general practices
2	Seek the opinions of key stakeholders involved in the SMR process about the ways in which AI approaches can be used to improve the process and identify what their requirements are for prescriber feedback systems.1.		
3	Identify potential barriers/facilitators to uptake and utilisation of AI-augmented SMRs and audit and feedback dashboards for clinicians.2.		
4	Curate structured clinical data from integrated records (general practice, hospital, and social care) from a variety of NHS Integrated Care Systems covering ∼11m population, adding more structured data from Natural Language Processing (NLP) of psychiatric narratives in Merseyside.	What combinations of diseases, medications, investigations and clinical contacts are associated with the greatest degree of adverse outcomes in patients with high risk of harm (frailty, co-existent mental and physical health problems and complex multimorbidity)?	A pipeline of structured and unstructured care data into multimorbidity (AI) research.
5	Use AI approaches and statistical methods to identify patterns and clusters of conditions, medications, tests, and clinical contacts preceding adverse events across three target groups then build the patterns into biostatistical causal inference and prediction of (clustered) clinical outcomes.		An AI framework for identifying those most at risk of problematic polypharmacy and for discovering disease trajectories that should trigger high priority SMRs
6	Develop visualisation methods for longitudinal summaries of multi-provider care records overlain with risk trajectories, combined with key features from AI-learned patterns/structures and clinical guidelines.	Can patient journeys over time and across care providers be adequately visualised in the context of clinical guidelines and be enriched with causal inference methods?	Novel visualisations of patient journeys enhancing medication reviews
7	Co-design a prototype tool, through iterative review and refinement of feedback systems – participating clinicians, who undertake SMRs will participate in “think-aloud” studies of the protype tool and identify positive and negative features of the tool which will allow the iterative improvement of the prototype (co-developed with patient and public representatives).	Can a learning system be created that incorporates the needs of prescribers alongside the key high-risk trajectory indicators?	Integration of outputs 1 – 4 to produce a clinically useful learning healthcare system, co-developed by the end users and supporting the delivery of SMRs by GPs and pharmacists and be accessible to patients/carers
8	Refine the later prototypes through user-group feedback and, through two workshops, to explore further the perceived strengths and weaknesses and thus the implementability of the system.		

The 2021 NHS Overprescribing Review sets out a plan to reduce overprescribing and improve patient safety. The report identifies a key evidence gap, recommending new research to support safe and appropriate prescribing, specifying research to ensure digital systems and records make structured medication reviews a simple task.^2^ DynAIRx directly addresses this important evidence gap.

In the longer term (DynAIRx 2) we will build multimorbidity decision support on DynAIRx visualisations and outcome predictions, for use in consultations.

## Supplemental Material

Supplemental Material - The DynAIRx Project Protocol: Artificial Intelligence for dynamic prescribing optimisation and care integration in multimorbidity Click here for additional data file. Supplemental Material for The DynAIRx Project Protocol: Artificial Intelligence for dynamic prescribing optimisation and care integration in multimorbidity by Lauren E Walker, Aseel S Abuzour, Danushka Bollegala, Andrew Clegg, Mark Gabbay, Alan Griffiths, Cecil Kullu, Gary Leeming, Frances S Mair, Simon Maskell, Samuel Relton, Roy A Ruddle, Eduard Shantsila, Matthew Sperrin, Tjeerd Van Staa, Alan Woodall and Iain Buchan in Journal of Multimorbidity and Comorbidity
