# Supplementary material for: The DynAIRx Project Protocol: Artificial Intelligence for dynamic prescribing optimisation and care integration in multimorbidity
Source: J Multimorb Comorb. 2022 Dec 15;12:26335565221145493. doi: 10.1177/26335565221145493 (PMC9761229; doi:10.1177/26335565221145493)
Supplement: Supplemental Material - The DynAIRx Project Protocol: Artificial Intelligence for dynamic prescribing optimisation and care integration in multimorbidity [file sj-pdf-1-cob-10.1177_26335565221145493.pdf]

**Appendix 1: Health care Practitioner Interview Topic Guide**

The purpose of these one-on-one scoping interviews with key stakeholders is to draw out some common themes on challenges to undertaking structured medicines reviews that can be explored in greater depth. Accordingly, the initial scoping interviews will be broad in their scope.

Background: I will give a short (5 minute) overview about the Overprescribing review and structured medication reviews to introduce the topic.

I will show some example dashboards and Prescriber Feedback (from other projects, not specific to SMRs) and introduce the potential opportunity that AI presents to augment SMRs.

The following issues will then be addressed/explored:

**Topic 1: How are Structured Medication Reviews currently being undertaken where you work and how long do they take?**

What sort of services are in place? How long are the appointments allocated versus how long do they actually take per patient? Who does them? What is the process for escalation?

**Probe**: Why are SMRs performed in your workplace? What are the first steps? How do you decide in your setting who should be prioritized? Is it based on number of drugs? Complexity? Prescribing indicators? Frailty? Who does the prioritization? Can you give examples of the decisions you take? What leads you to arrive at each type of decision?

**Topic 2:** What do you consider the top priority target medication challenges relating to key multimorbidity groups (older people with frailty; co-existing physical and mental health problems; complex multimorbidity and potentially problematic polypharmacy)?

**Probe**: What are the risky medication combinations that present the biggest challenges? What are the hardest medications to withdraw? How many (relevant) diagnoses do they tend to have? How many different medications are they on? Typical/min/max.

**Topic 3: What data do you need to undertake effective Structured Medication Reviews efficiently?**

**Probe**: What digital resources do you need to review (letters, blood results) What are the main data gaps that would aid decision making (e.g. discharge letters and poor information flow from secondary to primary care)What information do you need about each diagnosis? What information do you need about each medication? (E.g., start date, dose, frequency, etc.) What other information do you need to know about a patient (age, etc.)

**Topic 4**: What kind of digital tools do you think would be most helpful for assisting efficient SMRs?

What tools are used currently – why – what are the benefits, drawbacks?

To prompt this I could show examples of currently available deprescribing resources and some idea of how long they take to implement (Drug burden index, BNF interaction checker)

**Topic 5**: What are likely barriers/facilitators to uptake and utilisation of AI(-augmented) tools?

What would be a sign to you that the intervention is having the desired effect?

**Probe**: What does success look like (number of reviews undertaken? most high-risk people prioritized?)

What outcomes would you like to see to show it was worth continuing to keep using it? What would put you off? How would you feel about being involved in trying out the intervention during the development phase, in order to feedback and improve it?

Any further areas of discussion:

- Do you have anything further to add from what we have discussed today?

**Appendix 2: Health care Practitioner Focus Group Topic Guide**

The contents of this focus group guide will be informed by the findings of the ongoing one-on-one scoping interviews with key stakeholders so may be subject to some changes in content.

Background: I will give a short (10 minute) presentation about the Overprescribing review, structured medicines reviews and the themes that have emerged from scoping interviews with key stakeholders about the possible barriers/facilitators to effective SMRs

I will show some example dashboards and Prescriber Feedback (from other projects, not specific to SMRs) and introduce the potential opportunity that AI presents to augment SMRs.

The following issues will then be addressed/explored:

**Topic 1:** How are Structured Medication Reviews currently being undertaken, by whom and where, and how long do they take?

What sort of services are in place? How long are the appointments allocated versus how long do they actually take per patient? Who does them? What is the process for escalation?

**Probe**: What are the first steps? What digital resources do you need to review (letters, blood results) What are the main data gaps that would aid decision making (e.g. discharge letters and poor information flow from secondary to primary care)

How do you decide in your setting who should be prioritized? Is it based on number of drugs? Complexity? Prescribing indicators? Frailty? Who does the prioritization?

**Topic 2**: What data do prescribers and practitioners involved in reviewing medications need to undertake effective Structured Medication Reviews efficiently?

What do you the participants think about the suggestions and themes that have merged from initial scoping interviews?

**Probe**: What do they agree with? What do they disagree with? Specifically focusing on the notion of AI to aid digital summarising of patient trajectories of risk – what are perceptions/ideas about the role of an AI-intervention?

**Topic 3**: What do participants consider the top priority target medication challenges relating to key multimorbidity groups (older people with frailty; co-existing physical and mental health problems; complex multimorbidity and potentially problematic polypharmacy)?

**Probe**: What are the risky medication combinations that present the biggest challenges? What are the hardest medications to withdraw?

**Topic 4:** What kind of digital tools do they think would be most helpful for assisting efficient SMRs?

What tools are used currently – why – what are the benefits, drawbacks?

To prompt this I could show examples of currently available deprescribing resources and some idea of how long they take to implement (Drug burden index, BNF interaction checker)

**Topic 5**: What are likely barriers/facilitators to uptake and utilisation of AI(-augmented) tools?

What would be a sign to them that the intervention is having the desired effect?

**Probe**: What does success look like (number of reviews undertaken? most high-risk people prioritized?)

What outcomes would they like to see to show it was worth continuing to keep using it? What would put them off? How would participants feel about being involved in trying out the intervention during the development phase, in order to feedback and improve it?

Any further areas of discussion:

- Do any of the participants have anything further to add from what we have discussed today?
